# The ABCDE approach to difficult weaning from venoarterial extracorporeal membrane oxygenation

**DOI:** 10.1186/s13054-022-04089-8

**Published:** 2022-07-15

**Authors:** Christiaan L. Meuwese, Daniel Brodie, Dirk W. Donker

**Affiliations:** 1 Department of Intensive Care Medicine, Erasmus Medical Center, PO Box 2040, 3000 CA Rotterdam, The Netherlands; 2 Department of Cardiology, Erasmus Medical Center, PO Box 2040, 3000 CA Rotterdam, The Netherlands; 3 Department of Medicine, Columbia University Medical Center, New York, USA; 4 Center for Acute Respiratory Failure, New York-Presbyterian Hospital, New York, USA; 5 Intensive Care Center, University Medical Center Utrecht, Utrecht, The Netherlands; 6 Department of Cardiovascular and Respiratory Physiology, TechMed Centre, University of Twente, Enschede, The Netherlands

**Keywords:** Extracorporeal membrane oxygenation, ECMO, Weaning failure

## Abstract

Venoarterial extracorporeal membrane oxygenation (VA ECMO) has been increasingly applied in patients with cardiogenic shock in recent years. Nevertheless, many patients cannot be successfully weaned from VA ECMO support and 1-year mortality remains high. A systematic approach could help to optimize clinical management in favor of weaning by identifying important factors in individual patients. Here, we provide an overview of pivotal factors that potentially prevent successful weaning of VA ECMO. We present this through a rigorous approach following the relatable acronym ABCDE, in order to facilitate widespread use in daily practice.

## Background

Venoarterial extracorporeal membrane oxygenation (VA ECMO) has become increasingly used for the urgent and potentially lifesaving support of patients with severe cardiorespiratory failure [1, 2]. This increase in popularity may be attributable to improving mortality rates and growing ease of VA ECMO initiation [3].

These encouraging developments are at least in part overshadowed by a considerable percentage (30–70%) of patients that can ultimately not be successfully weaned from VA ECMO support [4, 5]. When patients are not eligible for a durable ventricular assist device or heart transplantation, weaning failure implicitly means withdrawal of life-sustaining support. It is therefore apparent that great caution should be used to carefully review and optimize all reversible factors before VA ECMO extraction is performed. Naturally, factors that would clearly prevent successful weaning should be addressed before performing a weaning trial and inferring these conclusions.

To our knowledge, these factors have not been systematically summarized in a rigorous, clinical practice-oriented way. A comparable and well-recognized approach exists for patients who cannot be weaned from mechanical ventilation, known as ‘ABC,’ which forms the basis for weaning protocols from mechanical ventilation [6]. Given the imperative for such an overview to guide clinical practice in the setting of VA ECMO, we set out to summarize contributing factors and underlying disease-related reasons for VA ECMO weaning failure and provide a daily tool for a structured diagnostic and therapeutic approach.

## Failure to wean from VA ECMO

A multitude of studies have assessed the occurrence of VA ECMO weaning failure and its predisposing risk factors [5]. Throughout these studies, however, the definition of weaning failure varies considerably. Some authors have proposed that successful weaning should be defined as 30-day survival without (re)implantation of another mechanical support device [5, 7, 8]. Other groups have limited the critical time frame to 48 [9, 10] or even 24 h [11, 12] after decannulation.

A careful consideration of the exact definition of weaning success seems important as successful decannulation does not unequivocally translate into beneficial patient outcome [5, 13]. About 20 to 60 percent of patients still die after decannulation but before hospital discharge [4, 13]. This so-called ECMO gap seems largely explained by persistent multiorgan failure, adverse neurological events, bleeding, intercurrent infections (most notably pneumonia) and decompensated cardiac failure [14]. An additional 10 percent of patients ultimately die after hospital discharge but before 1-year follow-up [15]. This seems mainly attributable to underlying disease states, as well as the acquired frailty after a prolonged and complicated intensive care stay [16].

## Consequences of VA ECMO weaning failure

VA ECMO is intended to serve as a bridge to recovery in most cases. When recovery is not possible and a patient remains VA ECMO dependent, prognosis is poor or dependent on long-term therapeutic options [17]. A way out is only possible by implantation of a mechanical circulatory support (MCS) device, i.e., a left ventricular assist device (LVAD) or a biventricular assist device (BIVAD)), or urgent heart transplantation. Yet, these bridging steps are generally restricted to the most favorable prognostic scenarios while on ECMO and patient selection requires careful evaluation and weighing of specific prognostic factors [18–20]. In France, patients receiving VA ECMO are prioritized for cardiac transplant but their eligibility is confined to 12–16 days of extracorporeal support duration to optimize their waitlist and posttransplant mortality [20].

Patients who ultimately receive an LVAD in the setting of VA ECMO weaning failure have a 30-day survival of 77 percent which drops to about 50 percent after 1 year [18]. This last number approximates overall survival of VA ECMO-supported patients [15].

## The ABC concept as a systematic approach to VA ECMO weaning failure

**Fig. 1:**
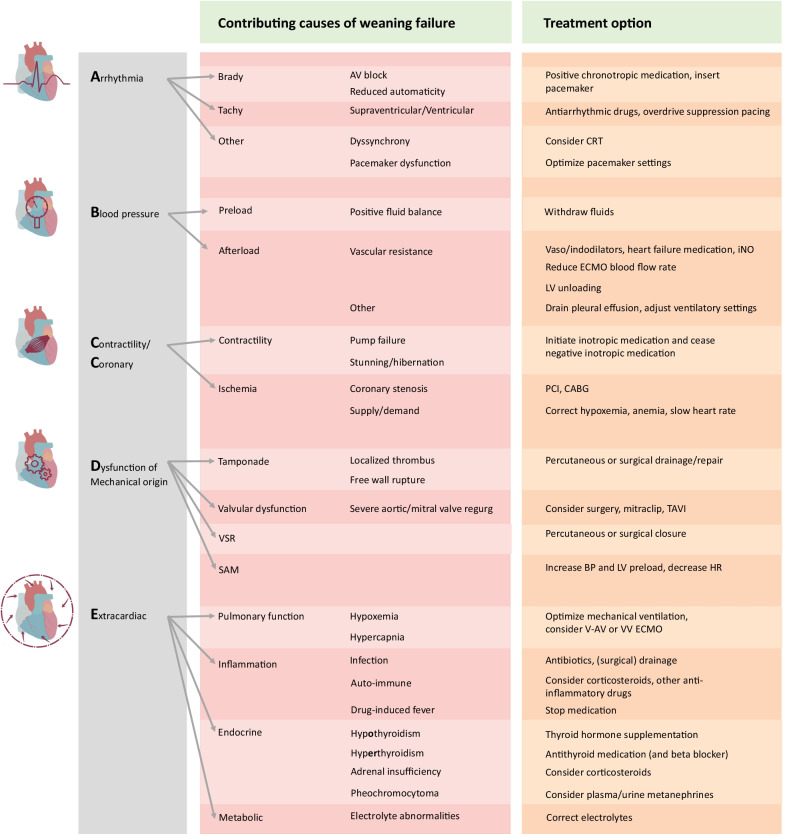
Contributing causes to VA ECMO weaning failure categorized according to the ABCDE concept. AV: atrioventricular. CRT: cardiac resynchronization therapy. iNO: inhaled nitric oxide. ECMO: extracorporeal membrane oxygenation. PCI: percutaneous coronary intervention. CABG: coronary artery bypass grafting. TAVI: transcatheter aortic valve implantation. VSR: ventricular septal rupture. SAM: systolic anterior motion of the mitral valve. BP: blood pressure. LV: left ventricle. HR: heart rate. V-AV: veno-arterial venous. VV: venovenous

A systematic and concise evaluation of all reversible factors may maximize weaning success rates and outcomes of VA ECMO-supported patients. As many different and potentially interrelated factors may be dynamically involved in each individual patient, a systematic approach to this critical process is necessitated. In the absence of definitive guideline recommendations and clear evidence concerning weaning from VA ECMO, the diagnostic and therapeutic recommendations outlined here are based on the practice experience and opinion of the authors and their critical appraisal of the literature. In Fig. 1, an overview of the various factors is provided. Depending on the specific case, the reader may find some factors of greater importance than others. Also, several factors may have already been assessed during an earlier phase of ECMO support. The practical approach described here is intended to provide a complete overview of factors that may contribute to persistent VA ECMO dependency. Naturally, this approach can also be extrapolated to patients who clinically deteriorate after decannulation.

### A: arrhythmias

Cardiac output can be significantly compromised by both bradyarrhythmias and tachyarrhythmias. In the weaning phase from VA ECMO, it seems advisable to strive for a normally conducted sinus rhythm with a heart rate of 60–100 beats per minute (bpm), whenever possible.

#### Bradyarrhythmias

From a conceptual perspective, a lowest possible heart rate seems ideal in recovering heart failure under VA ECMO support as it may limit myocardial mechanical loading, calcium (dys-)handling and overload, and metabolic demand directly. A sinus rhythm with a rate between 60 and 80 bpm may at times be acceptable during weaning from VA ECMO, especially in ischemic heart disease. When cardiac output is strongly compromised by the low heart rate itself, e.g., in the context of a relatively rate-independent, ‘*fixed’* stroke volume, temporary pacing at high normal rates (100 bpm) should be considered to facilitate weaning from VA ECMO. In such instances, chronotropism can be stimulated through adrenergic medications. A high-degree atrioventricular block may respond accordingly, while distal conduction block may become worse and also warrant temporary pacing.

#### Tachyarrhythmias

A high heart rate (> 100 bpm) is inherently related to a significant mechanical and metabolic myocardial burden and should therefore, especially in a recovering heart, be reduced as much as possible to a degree that still guarantees an acceptable native cardiac output.

Supraventricular (tachy-)arrhythmias, i.e., atrial fibrillation and flutter, are frequently encountered during critical illness and may be caused by a combination of cardiovascular failure and systemic inflammation in the setting of multiorgan failure [21]. A (tachy-)arrhythmia-induced cardiomyopathy can be the cause of severe cardiogenic shock requiring VA ECMO [22], but may also contribute to persisting VA ECMO dependency. It can do so by ablating the atrial kick, compromising passive ventricular filling and reducing myocardial contractility. A ‘*rhythm control*’ strategy seems preferable to assure the persistence of an ‘atrial kick.’ Yet, restoration of sinus rhythm does not necessarily result in the immediate recovery of mechanical forces and atrial stunning may persist for days. Therefore, a ‘*rate control*’ strategy may be equally valid when sinus rhythm cannot durably be achieved [22]. An arrhythmia-induced cardiomyopathy justifies an aggressive rate control strategy, including definite ablation of the atrioventricular node, and pacing [22].

Ventricular arrhythmias during VA ECMO support can arise due to a variety of factors which include electrolyte abnormalities (see section ‘E: extracardiac factors’), pro-arrhythmogenic side effects of medication, myocardial ischemia, novel structural abnormalities or complications (see section ‘D: dysfunction of mechanical origin’). The occurrence of ventricular arrhythmias mandates a thorough diagnostic work-up. Although ventricular arrhythmias may not immediately lead to hemodynamic compromise in the setting of extracorporeal support, they should immediately be attended to as the absence of native cardiac ejection may cause pulmonary ischemia, left-sided cardiac congestion and distention, pulmonary edema and thrombo-embolism. Depending on the underlying pro-arrhythmogenic mechanisms involved, temporary pacing at rates above the baseline heart rate that allowed ventricular arrhythmias to occur, possibly combined with anti-arrhythmic medications, may prevent recurrences. Also, catheter ablation should be considered, which may be safely performed during VA ECMO support [23].

#### Dyssynchronopathy

It is well appreciated that an altered atrioventricular and inter-ventricular dyssynchrony significantly impacts on heart failure severity and cardiac output. Cardiac output and efficiency may improve with cardiac resynchronization through atrioventricular and biventricular pacing [24]. From a mechanistic perspective, these insights from optimizing heart failure by chronic pacing may also prove beneficial in the setting of acute heart failure and VA ECMO support. Yet, the literature on optimal atrioventricular and biventricular pacing is scarce and limited to case reports and series and requires further study [25].

#### Diagnostic and therapeutic recommendations for A: arrhythmias

- Strive for sinus rhythm. When cardiac output depends on maintaining a very low stroke volume, consider aiming for heart rates around 100/min by means of medication or pacing.
- Aggressively correct ventricular arrhythmias and search for contributing factors.
- In severe dyssynchrony impairing cardiac performance, consider resynchronization therapy to enable weaning.

### B: blood pressures and loading conditions

A key aspect of heart failure management is to unload the heart by lowering preload and afterload, reduce myocardial metabolism and oxygen demand, and favorably influence the remodeling processes, while maintaining cardiac function and integrity. This important notion directly translates into optimal blood pressure management and is particularly important during VA ECMO support where the heart is additionally burdened by the non-physiologic, retrograde and continuous aortic flow of the extracorporeal circuit [26, 27]. Despite these challenges, the best prerequisites for weaning will be met through an optimization of ventricular preload and afterload.

**Fig. 2:**
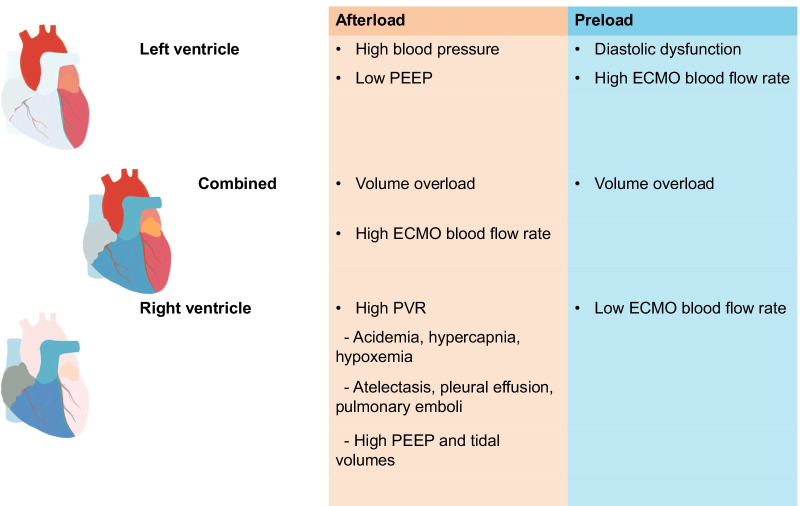
Modifiable factors influencing preload and afterload categorized by left and right ventricle. ECMO: Extracorporeal membrane oxygenation. PEEP: Positive end-expiratory pressure. PVR: pulmonary vascular resistance

Both preload and afterload are influenced by a wide array of factors, some of which are shared by both the left (LV) and right ventricle (RV) [27, 28]. These factors are summarized in Fig. 2. Preload of both ventricles is lowered by decreasing circulating blood volume. Left ventricular afterload is primarily determined by arterial blood pressure which in turn is greatly influenced by systemic vascular tone and VA ECMO blood flow. A lowering of the ECMO blood flow rate and blood pressure through decreasing the ECMO pump speed, inodilators or heart failure medication results in a more beneficial left ventriculo-arterial coupling. Adjunct left ventricular unloading should always be considered when aortic valve opening is incomplete or even absent; echocardiographic ‘smoke’ can be detected reflecting low blood flow in the LV, left atrium or ascending aorta; the pulmonary capillary wedge (PCWP) or left atrial pressure is significantly elevated (> 15 mmHg) or when pulmonary edema is present. Please note in this context that left atrial or ventricular dilatation merely represents cardiac geometry and does not necessarily translate into myocardial overload without considering cavity pressures.

The RV generally tends to be unloaded during VA ECMO support, which also renders it more challenging to assess its proper contractile performance. As such, right ventricular–pulmonary arterial coupling deserves particular attention as insufficient right ventricular function often forms a limiting step in the weaning process from VA ECMO. Pulmonary vascular resistance (PVR) should be optimized by striving for a low–normal partial pressure of CO_2_ and a high normal partial pressure of O_2_ in the arterial blood both promoting vasodilation. Also, inhaled nitric oxide (iNO) or other potent and selective pulmonary vasodilators can significantly aid to reduce the PVR.

#### Diagnostic and therapeutic recommendations for B: blood pressures and loading conditions

- Lower afterload as far as physiologically tolerable through the use of inodilators and possibly heart failure medications as well as by decreasing the ECMO pump speed.
  o For right ventricular unloading, consider aiming for physiological pH or mild alkalemia, normal partial pressures of arterial carbon dioxide and oxygen, and consider adding iNO.
  o For left ventricular unloading: a) lower blood pressure and ECMO blood flow rate as far as tolerated by end-organ perfusion and b) consider adjunct LV unloading.
- Lower preload as physiologically tolerated by decreasing the circulating blood volume through diuretics or continuous renal replacement therapy/ ultrafiltration. Strive for CVP < 12 and PCWP < 15 mmHg.

### C: contractility and coronaries

An adequate degree of contractile reserve and its proper stimulation to meet circulatory demands is a prerequisite for successful VA ECMO weaning. Myocardial contractility is influenced by many factors including an intact intra- and inter-cellular myocardial structure, provision of sufficient nutrients and oxygen, proper neuro-vegetative and neuro-humoral regulation, thyroid hormone signaling, and an adequate electrolyte homeostasis including cardiomyocyte calcium handling. Cardiac performance and myocardial integrity may also be negatively influenced by the effects of VA ECMO, an effect which seems primarily the consequence of alterations in cardiac loading conditions [29].

Therapeutic interventions improving native contractility should be aimed at optimizing cardiac loading conditions (see section ‘B: blood pressures and loading conditions’), myocardial oxygen consumption and initiating specific medication. No randomized clinical trials have evaluated the effects of inotropic medication on weaning success rates in the setting of VA ECMO. While observational studies in VA ECMO recipients suggested that epinephrine was associated with an increased mortality risk [30], administration of levosimendan was associated with a 1.6 times higher pooled odds for successful VA ECMO weaning [31]. In addition, a case series also suggested that initiation of phosphodiesterase-III inhibition (milrinone or enoximone), in conjunction with low-dose beta blocker administration, increased weaning success from VA ECMO [32]. Overall, inodilator therapy may have beneficial effects associated with an increased weaning success rate.

Further, coronary oxygen content and myocardial oxygen demand should be optimized as much as possible. Appropriate transfusion thresholds and hemoglobin targets for the setting of VA ECMO weaning failure are currently unknown.

#### Diagnostic and therapeutic recommendations for C: contractility and coronaries

- Consider the initiation of inodilator therapy and heart failure medication, while avoiding the potential negative sequelae of these drugs.
- In case of failure to wean, optimize oxygen content of arterialized blood. Please beware to measure arterial oxygen saturation on the right arm as this most consistently reflects coronary blood oxygenation.
- Reduce myocardial oxygen demand and reduce systemic oxygen extraction as much as possible by suppressing fever, work of breathing and shivering.
- When signs of myocardial ischemia exist and/or coronary status is unknown, consider performing a (repeat) coronary angiography. Revascularization should be pursued in most cases where severe coronary artery stenoses are found.

### D: dysfunction of mechanical origin

Several mechanical issues may cause cardiac dysfunction and prevent successful liberation from VA ECMO. These include cardiac tamponade, ventricular septal rupture (VSR), new onset severe mitral valve regurgitation (due to chordal rupture after myocardial infarction), and systolic anterior motion (SAM) of the anterior mitral valve leaflet with resulting severe mitral regurgitation and left ventricular outflow tract obstruction (LVOTO).

The occurrence of cardiac tamponade has been described in about 2 percent of patients supported with venovenous (VV) and VA ECMO [34]. Cardiac tamponade might develop secondary to reactive pericarditis, e.g., after myocardial infarction or heart surgery, perimyocarditis [35], free wall rupture [36], cardiac perforation during cannulation [34] or accumulation of thrombus in the pericardial sac after cardiac surgery. Recognition of cardiac tamponade may be obscured in the setting of VA ECMO as traditional signs are mitigated by the effects of a parallel extracorporeal circulation as created by the VA ECMO circuit. A clinical suspicion of cardiac tamponade in combination with pericardial effusion or thrombus should prompt drainage of a pericardial effusion. With weaning success rates and in-hospital survival rates approximating 82 and 73 percent, respectively, the prognosis of patients after drainage appears relatively good [34].

Ventricular septal rupture as a contributing factor to persistent shock during VA ECMO support might be clinically hard to distinguish from left and right ventricular failure due to myocardial infarction. Suspicion should be raised when new right ventricular dilatation or failure develops, a new loud murmur is appreciated, or pulmonary artery catheter-based measurements indicate a significant step-up in oxygen saturation and/or high cardiac output despite persistent low end-organ perfusion shortly after a myocardial infarction. Thirty-day mortality among patients with VSR under VA ECMO support was described as varying between 43 and 57 percent [37].

Clinically significant mitral valve regurgitation may be observed in severe left ventricular heart failure on a regular basis and may negatively influence the weaning process from VA ECMO. Also, device-related mitral valve regurgitation has been described as a complication of Impella support, which might be used in conjunction with VA ECMO for left ventricular unloading and as a facilitator for weaning from VA ECMO [38]. VA ECMO support for severe mitral valve regurgitation secondary to papillary muscle rupture has been described in 42 patients in the ELSO registry [37]. In-hospital survival approaches 43 percent [37], and therapeutic options include valvular repair or replacement [39] and percutaneous repair [40].

Patients with cardiogenic shock due to left ventricular outflow tract obstruction (LVOTO) as a result of hypertrophic cardiomyopathy [41] and stress-induced cardiomyopathy [42] have been successfully supported with VA ECMO. As LVOTO requires an increase in left ventricular afterload and preload, VA ECMO conveys beneficial effects on its severity [27]. For the same reason, the contribution of LVOTO to VA ECMO weaning failure might be underappreciated or even go unnoticed at high VA ECMO blood flow rates. Special attention should therefore be devoted to its presence during a weaning trial or when cardiogenic shock ensues shortly after decannulation. Percutaneous transluminal septal myocardial ablation, or surgical myectomy with or without mitral valve plasty/replacement must be considered when VA ECMO liberation depends on the presence of LVOTO and conservative measures fail [41].

#### Diagnostic and therapeutic recommendations for D: dysfunction of mechanical origin

- In case of failure to wean from VA ECMO, actively exclude a clinically significant degree of pericardial effusion/thrombus, the presence of a VSR, severe mitral valve regurgitation, and LVOTO.
- Note that the functional consequences of cardiac tamponade, VSR, and LVOTO might be masked by the inherent hemodynamic effects of VA ECMO.
- In case of a hemodynamically compromising degree of pericardial effusion/thrombus, consider drainage or surgical exploration prior to performing a weaning trial.
- In case of VSR, consider repair or percutaneous closure after an initial stabilization phase.
- In case of new onset severe mitral valve regurgitation, consider surgical valve repair/replacement or percutaneous repair.
- In case of LVOTO due to hypertrophic cardiomyopathy, consider alcohol septal ablation, surgical myectomy.

### E: extracardiac factors

Myocardial function is strongly influenced by a wide array of extracardiac factors which may be grouped into the following categories:

#### Mechanical ventilation

Most patients receiving VA ECMO require invasive mechanical ventilation as severe cardiogenic shock is often accompanied by overt respiratory insufficiency. Although VA ECMO may provide supplementary respiratory support, optimal management of mechanical ventilation remains indispensable and the impact of gas exchange depends on the degree of dual circulation and differential oxygenation present [43–45]. This includes appreciating heart–lung interaction, assuring adequate pulmonary mechanics and counteracting hydrostatic pulmonary edema, the latter being a common and serious complication of VA ECMO [46–48].

The current literature does not provide clear evidence to guide daily practice of mechanical ventilation during VA ECMO. Yet, pathophysiological considerations may aid in setting clinical goals that should be considered when aiming to optimally promote weaning from VA ECMO.

##### Positive end-expiratory pressure

Finding an optimal positive end-expiratory pressure (PEEP) requires weighing influences on right and left ventricular preload and afterload, while striving to minimize pulmonary edema and atelectasis. Generally, a high–normal PEEP around 10 cm H_2_O seems a fair compromise, while combined echocardiographic and hemodynamic monitoring of right and left ventricular geometry and function can aid in determining what appears optimal for the individual patient. This becomes particularly relevant when native pulmonary gas exchange becomes pivotal when the heart increasingly ejects blood originating from the pulmonary circulation into the ascending aorta and coronary arteries. Likewise, right ventricular function will benefit from a low–normal PVR in the absence of edema and atelectasis.

##### Lung-protective ventilation

VA ECMO-related lung injury might be important and mediated by different mechanisms and clinical entities including not only hydrostatic pulmonary edema, but also inflammation, alveolar hemorrhage, thrombo-embolism, and ischemia, the latter being sequelae of reduced transpulmonary blood flow during periods of high extracorporeal blood flow rates [49]. This all implies that a lung-protective ventilatory strategy, as is widely accepted for patients with the acute respiratory distress syndrome (ARDS), should be the goal, while gas exchange should suffice to guarantee a functional right–left shunt as inherent to VA ECMO. Toward the time of anticipated weaning from VA ECMO, patients should not be substantially dependent on the membrane oxygenator for gas exchange, but rather be mechanically ventilated to achieve adequate gas exchange.

#### Endocrine effects

Heart failure requiring ECMO support can occur in both hyperthyroidism [50] and hypothyroidism [51]. It is unknown to what extent thyroid hormone alterations could contribute to VA ECMO weaning failure. This is especially relevant since thyroid hormone alterations in the absence of disease in the hypothalamic–pituitaric–thyroidal axis (so-called *non-thyroidal illness*) are observed in a majority of critically ill patients [52]. In the absence of unequivocal evidence, we would advocate to consider correcting severely altered thyroid hormone levels in patients who experience VA ECMO weaning failure.

Adrenal insufficiency can precipitate heart failure [53, 54] and even necessitate VA ECMO support in severe cases [51, 55]. It is unknown to what extent (relative) adrenal insufficiency plays a role in ECMO weaning failure. A thorough endocrinological work-up seems imperative when no apparent explanation is found for high vasopressor needs and typical features of adrenal insufficiency exist. In exceptional cases, a pheochromocytoma can also explain cardiogenic shock necessitating ECMO support through catecholaminergic stress [56].

#### Inflammatory conditions

Development of infection has been described in 14 to 65 percent of ECMO-supported patients [57] where chances of contracting an infection seem to accumulate as ECMO support duration lengthens [58]. In a selection of patients who were supported longer than 48 hours, the majority of infections were characterized as ventilator-associated pneumonia (VAP: 55%), followed by blood stream infections (18%), cannula infections (10%), and mediastinitis (11%). Some studies have suggested that acquiring an infection during ECMO support is associated with a significantly increased risk for weaning failure and in-hospital mortality [59].

#### Electrolyte disturbances

Transient episodes of congestive heart failure [60, 61] and cardiogenic shock [62] have been reported in patients with overt hypocalcemia. Also, historical studies have indicated improvements in cardiac output and mean arterial blood pressure after intravenous calcium infusion in patients during, or shortly after, cardiopulmonary bypass [63, 64]. The effects of serum calcium concentrations and calcium infusion on myocardial function during VA ECMO support have not been evaluated. The arrhythmogenic risk seems to be augmented by severe hypokalemia and hypomagnesemia [65]. Hyperkalemia, on the other hand, may cause serious conduction disturbances and severe bradycardia.

#### Diagnostic and therapeutic recommendations for E: extracardiac factors

- Aim for ventilator settings that optimize cardiac loading conditions, which should also improve right–left ventricular interdependence.
- In case of an active infection, consider repeating a weaning trial after adequate source control and antibiotic treatment. If delay is not possible, suppress fever and shivering as much as possible.
- In case of overt hypothyroidism, initiate thyroid hormone supplementation.
- When suspecting adrenal insufficiency, perform a thorough endocrinologic work-up and initiate mineralocorticoid and corticosteroid hormone supplementation, as indicated.
- In case of insufficient ventricular systolic performance, consider optimizing serum calcium levels (> 1.0 mmol/L).
- High–normal potassium (> 4.0 mmol/L) and magnesium (> 1 mmol/L) levels should be pursued.

After having discussed all the different factors, we provide a checklist of examinations (Table 1) which can be used in a patient who is difficult to wean from VA ECMO support. The order of relevance of these different examinations may differ, depending on the specific case.

**Table 1:** Checklist of examinations and findings in a patient who is difficult to wean from VA ECMO support

Examination	Signs	Possible diagnosis
Clinical signs	Loud systolic murmur	VSR, MR, or LVOT gradient
	Pulsus paradoxus	Cardiac tamponade
Telemetry	Bradycardia, (supra)ventricular tachycardia	Arrhythmia/conduction disorder
ECG	ST segment/T wave abnormalities	Myocardial ischemia
	Specific arrhythmia-related signs	Arrhythmia
	PQ interval and QRS width	Conduction disorder/dyssynchrony
Laboratory values	Troponin, CK-MB	Myocardial ischemia
	ABG, ScVO_2_, SmVO_2_	Pulmonary gas exchange, oxygen supply, and demand balance
	Hemoglobin levels	Oxygen supply
	Electrolytes	Electrolyte disturbances, adrenal insufficiency
	TSH, fT4, fT3	Thyroid dysfunction
	CRP, PCT	Infection, inflammation
Echocardiography	RV and LV function and VTI	Impaired contractility
	Regional wall motion abnormalities	Myocardial ischemia, dyssynchrony
	Valvular regurgitation and its severity	Severe MR or AR
	Dynamic pressure gradient LVOT and SAM	LVOT gradient
	Pericardial effusion/thrombus	Cardiac tamponade
Pulmonary artery catheter	Elevated PA pressures/PVR	High RV afterload
	Elevated CVP	Cardiac tamponade
	Elevated CVP and PCWP	Fluid overload/decompensation
	Significant step-up in oxygen saturation (from RA to PA)	VSR
X-ray/ CT scan	Atelectasis	RV afterload
	Pleural effusion Pneumonia	RV afterload
	Pulmonary embolism	RV afterload

ABG: Arterial blood gas analysis. AR: Aortic valve regurgitation. CRP: C-reactive protein. CVP: Central venous pressure. CK-MB: Creatine kinase muscle-brain fraction. MR: Mitral valve regurgitation. PCWP: Pulmonary capillary wedge pressure. RA: Right atrium. PA: Pulmonary artery. PVR: Pulmonary vascular resistance. PCT: Procalcitonin. SAM: Systolic anterior motion of the mitral valve. ScVO_2_: Central venous oxygen saturation. SmVO_2_: Mixed-venous oxygen saturation. TSH: Thyroid-stimulating hormone. fT4/ fT3: Thyroid hormones. LVOT: Left ventricular outflow tract. VTI: Velocity time integral. VSR: Ventricular septal rupture

## Future directions

The poor weaning success rates from VA ECMO today underscore the necessity to focus our efforts on a variety of factors that may improve future outcomes, including:

- It is imperative that a universal definition of VA ECMO weaning failure be agreed upon to maximize efficiency and comparability of relevant studies.
- Large-scale observational studies with high-quality and detailed data incorporating repeated measures for each patient are necessary for: (1) improving patient selection, (2) identifying predictors for weaning failure and successful LVAD implantation, and (3) the assessment of risk factors and their contribution to weaning failure.
- Finally, there is a clear unmet need for randomized clinical trial data investigating different therapeutic interventions which could improve weaning success. These include (1) optimal timing of VA ECMO extraction, (2) the effects of LV unloading devices, and (3) types and dosages of inodilator agents as facilitators of weaning from VA ECMO.

## Conclusions

While the number of VA ECMO applications has surged in recent years, many patients still cannot be weaned from VA ECMO support and 1-year mortality remains approximately 50 percent. Here, we have provided an overview of pivotal factors that potentially prevent successful weaning of VA ECMO. A structured approach (ABCDE) and evaluation could help to identify factors of importance in a specific patient. Future work should expand our understanding of VA ECMO weaning failure and identify therapeutic interventions to optimize these factors and improve weaning success rates.

## Data Availability

Not applicable.

## Abbreviations

ARDS: Acute respiratory distress syndrome
BIVAD: Biventricular assist device
Bpm: Beats per minute
CO2: Carbon dioxide
ECMO: Extracorporeal membrane oxygenation
ELSO: Extracorporeal Life Support Organization
iNO: Inhaled nitric oxide
LVAD: Left ventricular assist device
LVOTO: Left ventricular outflow tract obstruction
MCS: Mechanical circulatory support
PCWP: Pulmonary capillary wedge pressure
PEEP: Positive end-expiratory pressure
PVR: Pulmonary vascular resistance
SAM: Systolic anterior motion of the mitral valve
VA: Venoarterial
VAP: Ventilator-associated pneumonia
VSR: Ventricular septal rupture
VV: Venovenous
LV: Left ventricle
RV: Right ventricle
O2: Oxygen
